# Valsalva Retinopathy Presenting as Subretinal Hemorrhage

**DOI:** 10.1155/2024/4865222

**Published:** 2024-04-05

**Authors:** Lívia Conci, Eliza Pereira, Samia Navajas, Epitacio Silva Neto, Sérgio Pimentel, Leandro Zacharias

**Affiliations:** Department of Ophthalmology, University of Sao Paulo, Sao Paulo, Brazil

## Abstract

**Purpose:**

To describe a case of subretinal hemorrhage due to the Valsalva maneuver in a patient with no underlying chorioretinal disease.

**Methods:**

History and clinical examination, optical coherence tomography (OCT), fluorescein, and indocyanine green angiography.

**Results:**

We report a case of a 35-year-old man with a 4-day history of central vision loss in the left eye (OS) after a vomiting episode. His best-corrected visual acuity was 20/200 in OS. Fundus examination revealed a subretinal hemorrhage in the posterior pole, associated with a small preretinal hemorrhage in the superotemporal arcade. OCT confirmed the presence of a thick submacular hemorrhage and a focal hemorrhage beneath the inner limiting membrane along the superotemporal arcade. The patient was submitted to pars plana vitrectomy (PPV), subretinal injection of tissue plasminogen activator (tPA), and air tamponade on the following day. Most of the submacular hemorrhage was displaced, resulting in a satisfactory visual outcome (BCVA = 20/30 after 1 month of surgery). Fluorescein and indocyanine green angiography excluded conditions such as retinal arterial macroaneurysm, polypoidal chorioretinopathy, and choroidal neovascularization.

**Conclusion:**

Although rare, Valsalva retinopathy may present with submacular hemorrhage in a patient with no underlying chorioretinal disease. PPV and subretinal tPA injection may provide a good visual outcome.

## 1. Introduction

First described by Duane in 1972, Valsalva retinopathy (VR) is a clinical entity characterized by preretinal hemorrhage and sudden visual decrease [1]. It occurs secondary to Valsalva maneuvers such as physical activities, vomiting, constipation, or coughing [2]. The forced expiratory effort against a closed airway leads to abrupt elevation of intrathoracic and venous pressures, resulting in retinal capillary rupture [1]. This disorder usually has unilateral involvement, and carries a good prognosis, with spontaneous hemorrhage regression [2].

The most common site of bleeding is beneath the inner limiting membrane (ILM), but subhyaloid, intraretinal, and vitreous hemorrhage may also be present [3]. Subretinal hemorrhage is a sight-threatening condition, related to photoreceptor toxicity and mechanical blockage of oxygen and nutrients from the choriocapillaris [4, 5]. Association between subretinal hemorrhage and VR is very rare, and it has been reported previously in few cases [6–9]. In most of them, underlying retinal diseases were described, such as retinal arterial macroaneurysm [7], polypoidal chorioretinopathy [8], and high myopia [9].

Herein, we report a case of subretinal hemorrhage due to the Valsalva maneuver in a patient with no underlying chorioretinal disease. As it was fovea-involving bleeding, we performed pars plana vitrectomy (PPV) with tissue plasminogen activator (tPa) subretinal injection. Subretinal hemorrhage was displaced, and a good visual outcome was achieved.

## 2. Case Presentation

A healthy 35-year-old man presented with a 4-day history of sudden central vision loss in the left eye (OS) immediately after a vomiting episode. He denied any previous ocular history. His best-corrected visual acuity (BCVA) was 20/20 in the right eye (OD) and 20/200 in the OS. Anterior biomicroscopy was unremarkable, and intraocular pressure was normal in both eyes (OU). No vitreous cells were noted, and pupillary reflexes were normal.

**Figure 1:**
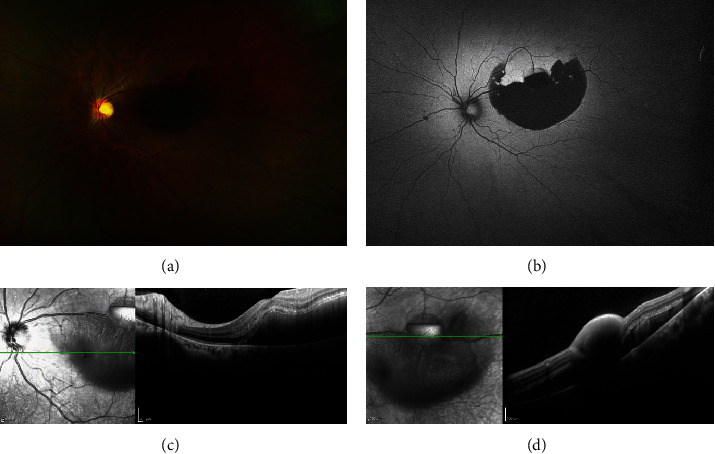
Multilayered retinal hemorrhages in the left eye. (a) Color fundus photograph (CFP) showed subretinal hemorrhage in the macular region, preretinal hemorrhage in the superotemporal arcade, and intraretinal flame-shaped hemorrhages around the optic disc. (b) Multilayered retinal hemorrhages had decreased autofluorescence sign. (c) Macular SD-OCT scan confirmed a thick intermediate-reflectivity hemorrhage in the subretinal space. (d) SD-OCT scan along the superotemporal arcade showed a shadowing effect of the preretinal hemorrhage located beneath the ILM.

In OS, a color fundus photograph (Daytona Ultra-widefield Retinal Imaging, Optos Inc., Marlborough, United States) revealed subretinal hemorrhage in the posterior pole associated with a small oval-shaped preretinal hemorrhage in the superotemporal arcade, two small flame-shaped hemorrhages around superior half of the optic disc, and few intraretinal hemorrhages (Figure 1(a)). Autofluorescence (Daytona Ultra-widefield Retinal Imaging) revealed that hemorrhages were hypoautofluorescence (Figure 1(b)). On spectral-domain optical coherence tomography (SD-OCT) (Spectralis, Heidelberg, Germany), the central macular thickness was 296 *μ*m in the OD and 594 *μ*m in the OS. Macular scanning confirmed the presence of a thick submacular hemorrhage in the OS, shadowing the choroid structures right below it (Figure 1(c)). SD-OCT scan along the superotemporal arcade showed a focal hemorrhage beneath the ILM with a shadowing effect of the underlying retina (Figure 1(d)). Fundus examination of the right eye was unremarkable.

**Figure 2:**
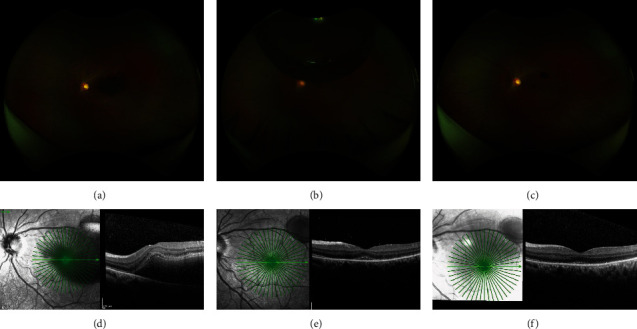
Sequential pre- and postoperative follow-up of the left eye. (a, d) Preoperative CFP and SD-OCT demonstrated subretinal hemorrhage in the macular region. (b, e) Five days after surgery, BCVA was 20/100. (b) CFP showed air bubbles in the vitreous cavity and subretinal hemorrhage displacement to the inferior equator and periphery. (e) SD-OCT confirmed submacular hemorrhage displacement, with only a tiny hyperreflective material beneath the fovea. (c) Ten days after vitrectomy, BCVA improved to 20/40. There was no air in the vitreous cavity, and subretinal hemorrhage was located at the inferior periphery. (f) SD-OCT did not demonstrate any submacular hemorrhage at this point.

Clinical examination and imaging results led us to diagnose VR associated with subretinal hemorrhage. On the following day, the patient was submitted to PPV, subretinal injection of tPA, and air tamponade to obtain a subretinal hemorrhage displacement outside the macula towards the inferior equator. This surgery was performed following the steps: after performing a 23-gauge PPV, 25 *μ*g tPA in 0.1 ml was injected in the subretinal space using a 38-gauge subretinal infusion needle (MedOne, Sarasota, FL), without ILM peeling, followed by air-fluid exchange. The patient remained in reading position (head at a 45° angle to the ground) for 2 days after surgery. At this time, most of the submacular hemorrhage had moved to the inferior equator and periphery. Baseline multimodal evaluation and 5-day and 10-day sequential follow-up examination are demonstrated in Figure 2. The visual acuity was improved to 20/100, five days after surgery, and to 20/40, after ten days.

**Figure 3:**
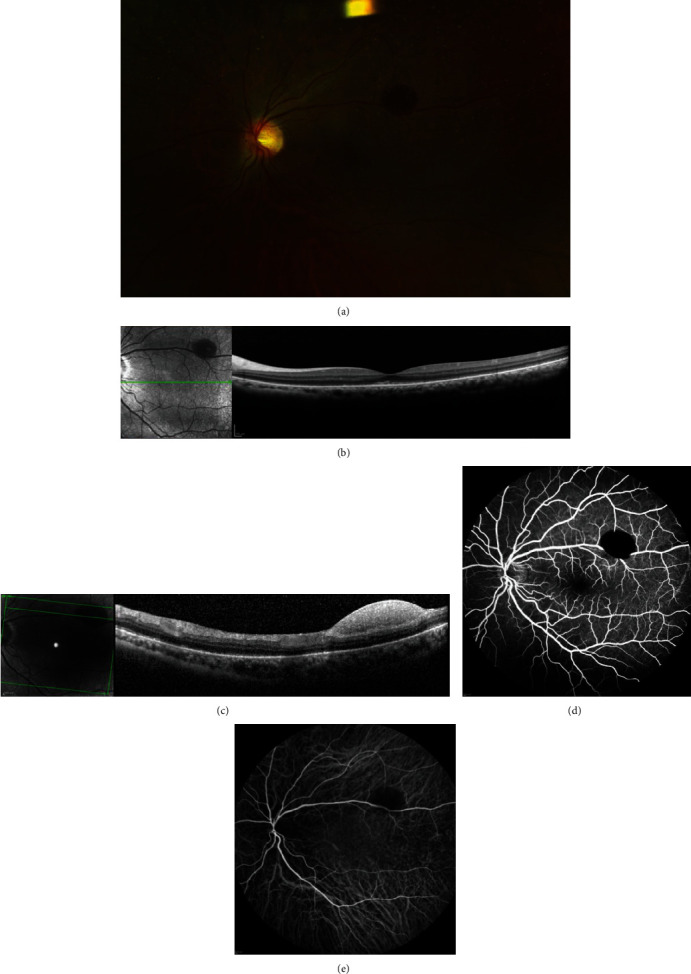
Multimodal evaluation of the left eye on the 30^th^ postoperative day. (a) CFP showed resolution of submacular hemorrhage. SD-OCT macular scan revealed (b) slight irregularity in the subfoveal ellipsoid zone and (c) persistence of sub-ILM hemorrhage in the superotemporal arcade. (d) Fluorescein angiography demonstrated blockage by the sub-ILM hemorrhage. There were no signs of capillary exclusion or retinal arterial macroaneurysm. (e) Indocyanine green angiography did not show any signs of polypoidal choroidal vasculopathy or choroidal neovascularization.

On the 30^th^ postoperative day, BCVA improved to 20/30 and submacular hemorrhage had totally disappeared (Figure 3(a)). SD-OCT macular scanning showed a slightly irregular ellipsoid zone (Figure 3(b)); central macular thickness decreased to 285 *μ*m. Sub-IML hemorrhage along the superotemporal arcade was still present (Figure 3(c)). Fluorescein angiography (FA) (Spectralis, Heidelberg, Germany) demonstrated blockage by the sub-ILM hemorrhage (Figure 3(d)). There were no signs of capillary exclusion or retinal arterial macroaneurysm. Indocyanine green angiography (ICGA) (Spectralis, Heidelberg, Germany) did not show any signs of polypoidal choroidal vasculopathy or choroidal neovascularization (Figure 3(e)).

## 3. Discussion

Submacular hemorrhage is an unusual presentation of VR. It has already been described in association with some chorioretinal disorders, such as retinal arterial macroaneurysm, polypoidal choroidal vasculopathy, and high myopia [7–10]. However, subretinal bleeding may be present as part of a natural course in all these conditions. A study conducted by Berrocal et al. evaluated retrospectively the clinical course of different presentations of submacular hemorrhage [6]. In one of the 31 eyes studied, submacular hemorrhage was related to VR. Few details about clinical history in this case were provided. Besides, the authors did not specify if subretinal bleeding was secondary just to the Valsalva maneuver or if it was associated with an underlying chorioretinal disease.

In our case, fluorescein and indocyanine green angiography excluded conditions such as retinal arterial macroaneurysm, polypoidal chorioretinopathy, and choroidal neovascularization. Moreover, our patient did not have high refractive errors, and that added to the history of vomiting led us to conclude that the subretinal hemorrhage was caused by the Valsalva maneuver. Our patient also presented a smaller region of sub-ILM hemorrhage, and we believe that subretinal bleeding resulted from blood dissection beneath the retina.

Classic VR can be managed in most cases by observation only [2]. On the other hand, subretinal hemorrhage needs different attention and an early approach. Toth et al. demonstrated in a cat model that there was photoreceptor damage 1 hour after the onset of submacular hemorrhage and significant outer retinal damage within 1 week [5]. Subretinal blood may damage the neurosensory retina in different ways: blockage of nutrient passage, shrinkage of outer retinal layers secondary to clot formation, and toxicity by certain substances, such as fibrin, iron, and hemosiderin [4, 5].

Although there are few reported cases of good evolution managed only with observation and intravitreal injections [10], vitrectomy with subretinal tPA is an established technique to manage submacular hemorrhage [11]. We performed vitrectomy with tPA subretinal injection and air five days after the onset of the symptoms. We chose not to peel the ILM, as the sub-ILM hemorrhage was extrafoveal. Most of the submacular hemorrhage was displaced outside the inferior arcade after the surgery, resulting in a satisfactory visual outcome (BCVA of 20/30 after 1 month of surgery).

In conclusion, VR may present with submacular hemorrhage in a patient with no underlying chorioretinal disease. PPV and subretinal tPA injection may provide a good visual outcome.
